# Age-Related Changes in the Cardiometabolic Profiles in Singapore Resident Adult Population: Findings from the National Health Survey 2010

**DOI:** 10.1371/journal.pone.0162102

**Published:** 2016-08-29

**Authors:** Tze Ping Loh, Stefan Ma, Derrick Heng, Chin Meng Khoo

**Affiliations:** 1 Department of Laboratory Medicine, National University Hospital, 5 Lower Kent Ridge Road, 119074 Singapore; 2 Epidemiology & Disease Control Division, Ministry of Health, 16 College Road, 169854 Singapore; 3 Public Health Group, Ministry of Health, 16 College Road, 169854 Singapore; 4 Department of Medicine, National University Hospital, 5 Lower Kent Ridge Road, 119074 Singapore; 5 Department of Medicine, Yong Loo Lin School of Medicine, National University of Singapore, 10 Medical Drive, 117597 Singapore; 6 Cardiovascular and Metabolic Disorders Program, Duke-NUS Graduate Medical School, 8 College Road, 169857 Singapore; Universita degli Studi di Milano, ITALY

## Abstract

We describe the centile trends of the blood pressure, glycemia and lipid profiles as well as renal function of a representative population who participated in the Singapore National Health Survey in 2010. Representative survey population was sampled in two phases, first using geographical/ residential dwelling type stratification, followed up ethnicity. 2,407 survey participants without any self-reported medical or medication history for diabetes mellitus, hypertension and dyslipidemia were included in this analysis. All biochemistry analyses were performed on Roche platforms. After excluding outliers using Tukey's criteria, the results of the remaining participants were subjected to lambda-mu-sigma (LMS) analysis. In men, systolic blood pressure increased linearly with age. By contrast, an upward inflection around late 40s was seen in women. The diastolic blood pressure was highest in men in the late 30s-50s age group, and in women in the late 50s-60s age group. All glycemia-related parameters, i.e. fasting and 2-hour plasma glucose and HbA1c concentrations increased with age, although the rate of increase differed between the tests. Total cholesterol and LDL-cholesterol concentrations increased with age, which became attenuated between the early 30s and late 50s in men, and declined thereafter. In women, total cholesterol and LDL-cholesterol concentrations gradually increased with age until late 30s, when there is an upward inflection, plateauing after late 50s. Our findings indicate that diagnostic performance of laboratory tests for diabetes may be age-sensitive. Unfavourable age-related cardiovascular risk profiles suggest that the burden of cardiovascular disease in this population will increase with aging population.

## Introduction

Cardiovascular disease is one of the major causes of morbidity and mortality globally. It is important to understand the trends and prevalence of its risk factors in the general population for current and future public healthcare planning. A National Health Survey (NHS) was conducted by the Ministry of Health in 2010 to obtain a representative view of the general health of Singapore resident adults [1]. In this study, we described the age-related changes of the blood pressure, glycemia and lipid profiles as well as renal function of a representative population who participated in NHS 2010 and had no self-reported history of these chronic diseases.

## Materials and Methods

### Study subjects

The data included this study were derived from the cross-sectional NHS conducted between 17 March 2010 and 13 June 2010. Detailed description of the survey design can be found in the official report [1], and received institutional ethics review board approval (Medical & Dental Board, Health Promotion Board, ref: 005/2009). The participants provided written informed consent for further analysis of the collected data. Briefly, the sampling of the survey participants was performed in two phases.

In phase 1, geographical zones and residential dwelling units were stratified and selected to yield a representative dwelling type distribution. In phase 2, a random sample of 7,696 individuals was selected from households identified in phase 1. Disproportionate stratified sampling was used to ensure sufficient sample size for reliable prevalence estimates of the minority ethnic groups such that the sample composed of 30% Chinese, 30% Malays, 30% Indians and 10% others. After excluding ineligible individuals for reasons such as pregnancy, recent childbirth or institutionalisation, death and overseas sojourn, 4,337 out of 7,512 eligible individuals aged 18 to 79 years participated in the survey (representing a participation rate of 57.7%). Of the survey participants, 2,407 individuals aged 18 to 79 years without any self-reported medical or medication history for diabetes mellitus, hypertension and dyslipidemia were included in our study (S1 Fig).

### Blood pressure measurement

Blood pressure (BP) was measured using mercury sphygmomanometer after adequate resting. Two BP readings were taken 30 seconds apart and averaged.

### Laboratory analysis

All blood samples were collected after an overnight fasting of at least ten hours, using standard phlebotomy procedure. The oral glucose tolerance test (OGTT) was performed by administering 75g of glucose (Trutol), and measurement of the plasma glucose concentration was repeated two hours later. Other laboratory parameters measured included glycated haemoglobin (HbA1c), fasting total cholesterol, high-density lipoprotein cholesterol (HDL), direct low-density lipoprotein cholesterol concentrations (LDL) and creatinine. The estimated glomerular filtration rate (eGFR) was calculated using the Chronic Kidney Disease Epidemiology Collaboration (CKD-EPI) equation [2].

### Statistical analysis

Tukey's criteria were applied at 10-year intervals to identify outlying values, defined as any value lying below the first quartile value minus 3 times the interquartile range, or above the third quartile value plus 3 times the inter-quartile range [3]. Outlying values identified in this manner were removed from subsequent analysis.

The remaining data were analysed using the LMS Chartmaker Light software (Medical Research Council, Cambridge, United Kingdom) to fit smoothed centile curves to reference data. Smoothed centile lines were generated for the 3rd, 10th, 25th, 50th, 75th, 90th and 97th percentiles. Details of this method have been described previously [4].

## Results

**Fig 1:**
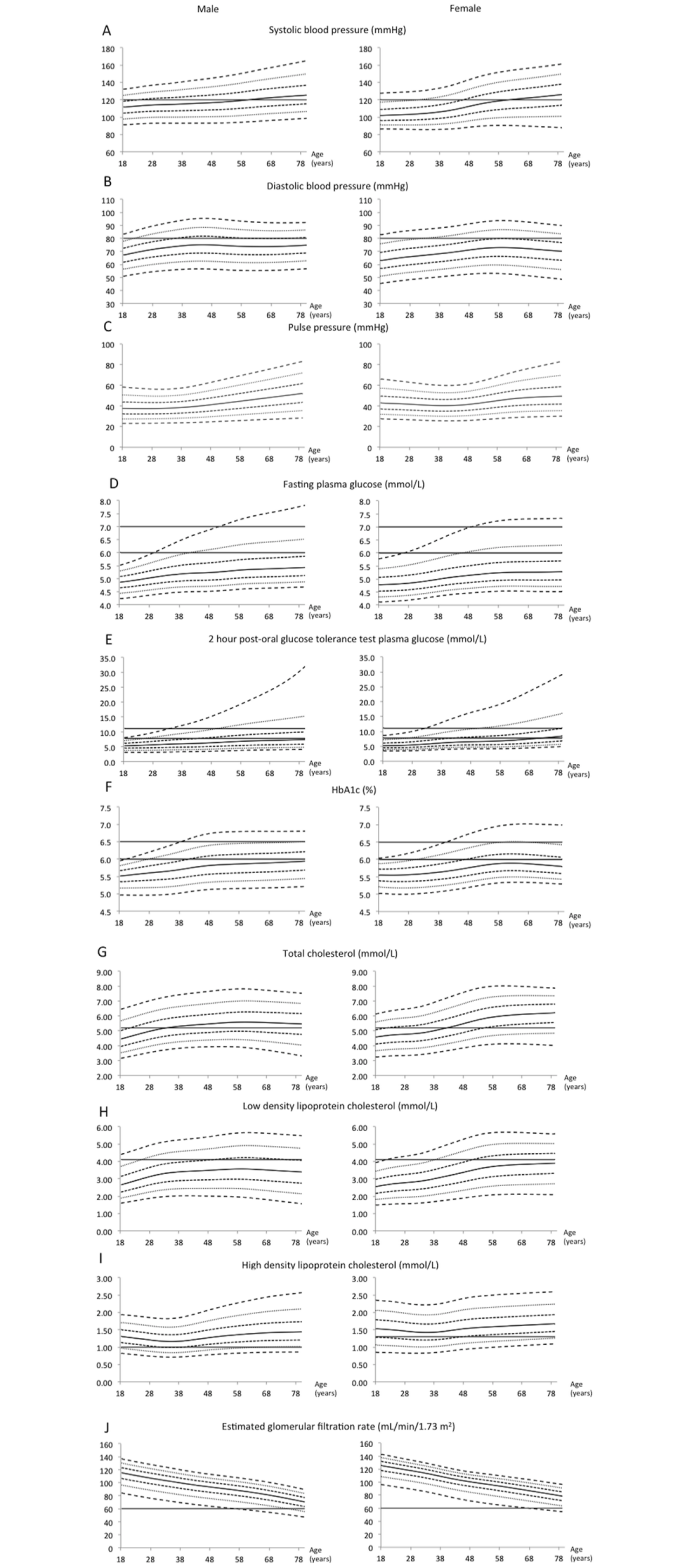
Centile charts of cardiometabolic parameters of subjects who participated in the Singapore National Health Survey 2010 and had no history of chronic illnesses. The broken lines represent (from bottom to top), the 3rd, 10th, 25th, 50th, 75th, 90th, 97th percentile lines of the survey population. The solid horizontal lines represent limits of normality or desired targets.

The average and standard deviation of the blood pressure, glycemia and lipid profiles as well as renal function of the male and female subjects are summarised in Table 1. The age distribution of the remaining subjects is shown in Table 2. The distribution of the laboratory parameters and BP measurements is shown in Fig 1. The participants included in this study did not have past medical or medication history for the diabetes mellitus, hypertension, dyslipidaemia or chronic kidney disease. Hence, those participants with parameters above the disease thresholds are considered undiagnosed subjects.

**Table 1:** The average and standard deviation of the blood pressure, glycemia and lipid profiles as well as renal function of the male and female subjects.

	Males		Females		t-test
Risk factors	Mean	SD	Mean	SD	p-values
Systolic blood pressure, mmHg	116	13	109	14	<0.0001
Diastolic blood pressure, mmHg	73	10	69	10	<0.0001
Pulse pressure, mmHg	43	41	12	11	<0.0001
Fasting plasma glucose, mmol/L	5.2	0.5	5.1	0.5	<0.0001
2-hour post-oral glucose tolerance test, mmol/L	6.2	1.9	6.3	1.8	0.12
HbA1c, %	5.7	0.4	5.7	0.3	0.02
Total cholesterol, mmol/L	5.30	0.96	5.18	0.97	0.003
Low density lipoprotein cholesterol, mmol/L	3.37	0.86	3.12	0.85	<0.0001
High density lipoprotein cholesterol, mmol/L	1.27	0.32	1.52	0.37	<0.0001
Triglycerides, mmol/L	1.32	0.63	1.01	0.50	<0.0001
Estimated glomerular filtration rate, mL/min/1.73 m^2^	98	15	107	15	<0.0001

**Table 2:** The age distribution of the subjects included in the analysis.

Age (in years)	Male	Female	Total
<30	273	363	636
30–40	316	370	686
40–50	288	341	629
50–60	146	163	309
>60	74	73	147
Total	1097	1310	2407

Older men and women tend to have higher BP than their younger counterparts. Systolic BP increased linearly with age in men. In women, a similar relationship was observed but with an upward inflection around late 40s. As such, systolic hypertension defined as systolic BP of 140 mmHg and above was present in 3% of men at the age of 38 years, and 10% at the age of 58 years. In comparison, similar diagnosis was present in 3% of women at older age of 48 years, and 10% at same age of 58 years. The diastolic BP was highest in men in their late 30s-50s, and in women in their late 50s-60s. However, the diastolic BP declined in older individuals.

All glycemia-related parameters, i.e. fasting and 2-hour plasma glucose and HbA1c concentrations, increased with age. Approximately 3% of the population had impaired fasting glucose (defined as having fasting plasma glucose of 6.0–6.9 mmol/L) at the age of 28 years, and more than 10% at the age of 40 years and above. Diabetes mellitus, defined as having fasting plasma glucose of 7.0 mmol/L and above, was present in 3% to 10% of the population around the age of 48 years.

More than 10% of the population had impaired glucose tolerance, defined as having 2-hour plasma glucose of 7.8–11.0 mmol/L, by the age of 30 years. Diabetes mellitus, defined as having 2-hour plasma glucose of 11.1 mmol/L and above, was present in 3% of the population by the age of 30 years, and more than 10% around the age of 48 years. Using HbA1c of 6.5% and above as the diagnostic threshold, diabetes mellitus was present in 3% of the population by age of 40 years, and between 3–10% by the age of 50 years.

Total cholesterol and LDL-cholesterol concentrations increased with age, which became attenuated between the early 30s and late 50s in men, and declined thereafter. In women, total cholesterol and LDL-cholesterol concentrations gradually increased with age until late 30s, when there was an upward inflection, plateauing after late 50s. Hypercholesterolemia defined as having fasting total cholesterol of 5.2 mmol/L and above was present in more than 50% of our study subjects around age of 40 years. In both men and women, HDL-cholesterol showed a U-shaped trend with age, where higher HDL-concentrations were observed in both younger and older subjects.

The eGFR declined continuously with age with a steeper gradient observed in men. As such, more than 10% of the men had stage 3 chronic kidney disease (i.e. eGFR <60 mL/min/1.73 m^2^) by late 70s.

## Discussion

Based on the results from NHS 2010, the cardiometabolic health profile of apparently healthy adults living in Singapore deteriorated with age. The systolic blood pressure, fasting and 2-hour plasma glucose, and LDL-cholesterol concentrations were higher in older men and women. In addition, there were gender differences in the age-related metabolic profiles, where systolic blood pressure, total and LDL-cholesterol concentrations in women were stable during the reproductive age, and only increased rapidly when they reached menopausal age.

Systolic BP increased with age but was attenuated in women during reproductive age, when female sex hormones such as estrogen and progesterone exert favourable effect on the vascular tone and renin-angiotensin system [5]. In contrast to systolic BP, we found that the diastolic BP was lower in older subjects, which could be due to increased large artery stiffness with increasing age [6,7].

Age-related stiffening of large arteries is associated with a decreased capacity of the elastic reservoir. During systole, greater proportion of stroke volume is being delivered to the periphery, reducing retention within the large arteries. At the beginning of diastole, the reduced residual blood volume and elastic recoil of the large arteries cause the diastolic pressure to decline [6,7]. The result of higher systolic but lower diastolic blood pressure is higher pulse-pressure with older age. In epidemiological studies, age-related reduction in diastolic blood pressure or higher pulse pressure has been shown to predict cardiovascular disease [6–8].

The reason for the increase in the fasting and 2-hour plasma glucose with age is likely due to an increase in the peripheral insulin resistance [9–11], age-related deterioration in β-cell function, physical inactivity, sarcopenia and obesity [12–14]. HbA1c also increases at a rate of 0.05–0.1% (0.55–1.09 mmol/mol) per decade, which is independent of the fasting and oral glucose tolerance [11]. The diagnostic specificity of HbA1c in detecting hyperglycaemia reduces with age. As a result, there have been calls to consider age-dependent hyperglycaemia and HbA1c diagnostic cutoff to maintain the diagnostic performance of this test [9,11].

In our study, we observed that total cholesterol concentration was lower in older men but higher in older women, and HDL-cholesterol concentration was higher in older subjects, which are consistent with previous cross-sectional and longitudinal studies [15–19]. These changes may be hormonally driven [20]. Total cholesterol rises steeply around menopause in women [21,22], and is primarily determined by the increase in LDL-cholesterol concentration.

Lower total cholesterol concentration in older individuals could be contributed by an age-related reduction in the food intake, cholesterol absorption, in cholesterol synthesis, and low LDL apo-B transport [17,19]. Interestingly, it has been suggested that lower total cholesterol concentration indicates frailty and can predict functional decline in older women [23]. Some of the observed changes in lipid profiles may be related to the change in sex hormone throughout adult life, where free testosterone decreases HDL in men while estradiol decreases LDL in women.

We observed that the eGFR was negatively associated with age in a near linear manner, with a steeper decline in men than women. This could be contributed by longer exposure to higher blood pressure in men throughout adulthood. The change in eGFR has been shown to be negatively associated with systolic BP, smoking, fibrinogen, and albumin/creatinine ratio [24,25]. On the other hand, high alcohol consumption in men and high physical activity in women were positively associated with eGFR.^24^ Whether the decline in the eGFR represents “normal” aging and its implication for diagnosis, progression and prognosis of chronic kidney and related diseases is still a subject of great debate [26,27]. The lower eGFR in the older population also indicate that they are more prone to acute kidney injury during an acute illness or dehydration.

It is important to bear in mind the cross-sectional nature of this study when interpreting any observed changes in the risk factors relates to the static population. These may give rise to potential confounding factors such as survival bias, where subjects with favourable longevity/survival factors (e.g. genotype or lifestyle) are over-represented in the study population, which may skew the data. A longitudinal study design would negate this limitation. Moreover, the exclusion of subjects with self-reported medical or medication history for diabetes mellitus, hypertension and dyslipidemia may result in underestimation of the trends observed. Another interesting future direction for research would be to examine race-related differences in these cardiovascular risk profiles, which was not assessed here due to the relatively small sample size.

In summary, our findings of unfavourable age-related cardiovascular risk profiles suggest that the burden of cardiovascular disease will increase as the Singapore resident population ages. The present data supports advocating health screening beginning at 40 years of age. However, further analysis on cost-effectiveness should be undertaken. Healthcare policy that emphasizes on primary prevention and early detection and optimal treatment of hypertension, diabetes and dyslipidemia will help to mitigate the increase of cardiovascular disease in the local population.

## Supporting Information

S1 Fig The number and characteristics of the subjects excluded from the analysis. (TIFF)
