# Supplementary figures and images for: Age-Related Changes in the Cardiometabolic Profiles in Singapore Resident Adult Population: Findings from the National Health Survey 2010
Source: PLoS One. 2016 Aug 29;11(8):e0162102. doi: 10.1371/journal.pone.0162102 (PMC5003348; doi:10.1371/journal.pone.0162102)

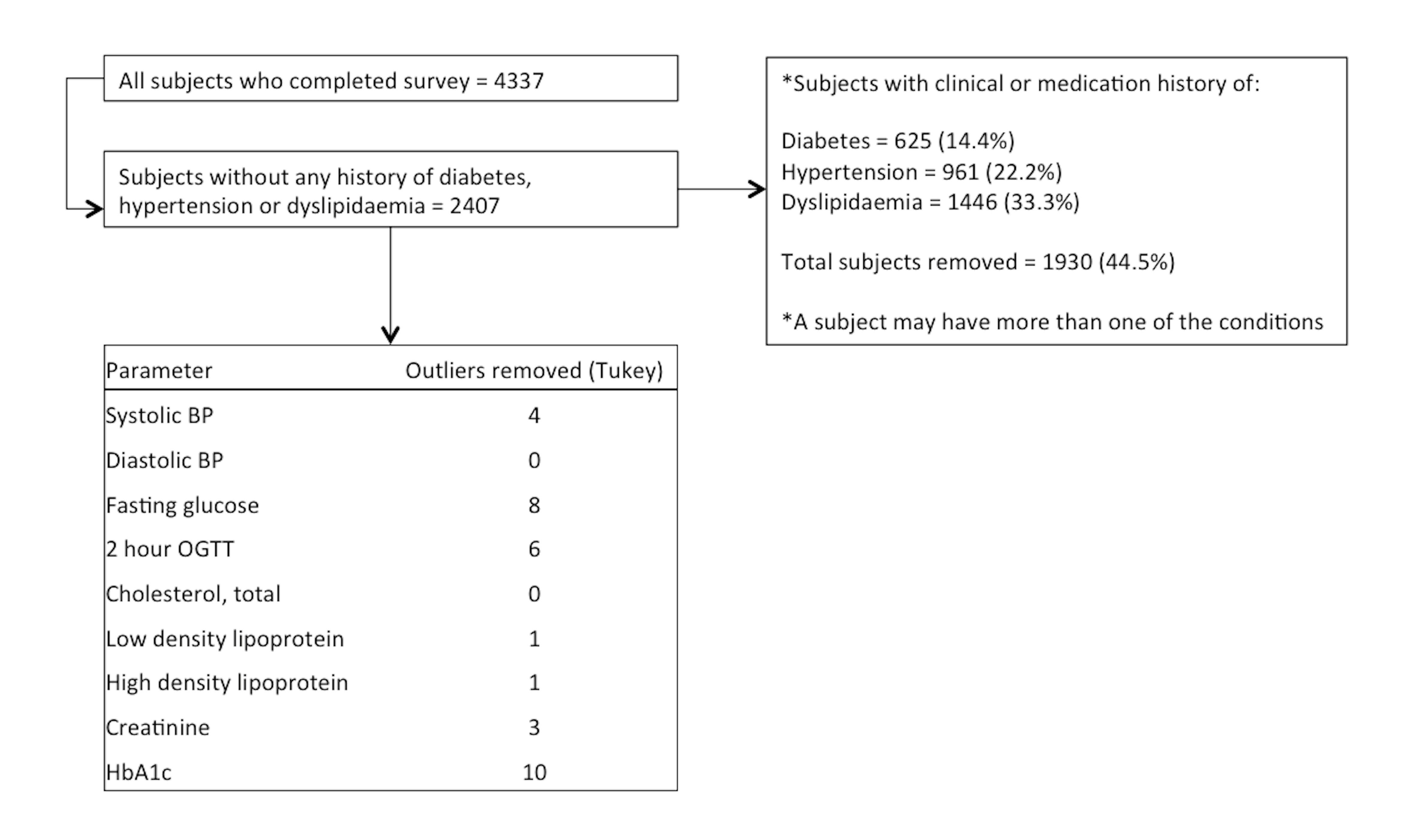

Supplement: S1 Fig — (TIFF) [file pone.0162102.s001.tiff]
